# Heme-Mediated Danger Signaling Suppresses Osteogenesis Through TLR4-Dependent Inhibition of β-Catenin Signaling

**DOI:** 10.3390/ijms27209305

**Published:** 2026-10-20

**Authors:** Xiaosong Xiang, Christiaan M. Suttorp, Peng Wang, Edwin M. Ongkosuwito, Marjon Bloemen, Johannes W. Von den Hoff, Frank A. D. T. G. Wagener

**Affiliations:** 1 Department of Dentistry, Orthodontics and Craniofacial Biology, Research Institute for Medical Innovation, Radboud University Medical Center, 6525 EX Nijmegen, The Netherlands; xiaosong.xiang@radboudumc.nl (X.X.); maarten.suttorp@radboudumc.nl (C.M.S.); edwin.ongkosuwito@radboudumc.nl (E.M.O.); marjon.bloemen@radboudumc.nl (M.B.); hans.vondenhoff@radboudumc.nl (J.W.V.d.H.); 2 Department of Rheumatology, Radboud University Medical Center, 6525 GA Nijmegen, The Netherlands; wangpeng4@jxutcm.edu.cn

**Keywords:** extracellular heme, osteogenesis, osteoblast differentiation, TLR4, AKT/GSK-3β/β-catenin signaling, Wnt signaling, osteoimmunology

## Abstract

Extracellular heme is a damage-associated molecular pattern (DAMP) released during hemolysis, hemorrhage, and tissue injury. Although its pro-inflammatory and cytotoxic effects are well established, its impact on osteoblast differentiation and bone formation remains incompletely understood. Here, we investigated the effects of extracellular heme on osteoblast differentiation and defined the signaling pathways involved. In MC3T3-E1 pre-osteoblasts, heme suppressed alkaline phosphatase (ALP) activity, calcium deposition, matrix mineralization, and expression of osteogenic markers, including Runx2 and Sp7 at day 3, Col1a1 and Alpl at day 7, and Bsp and Bglap at day 10, without affecting cell viability. Mechanistically, heme reduced AKT phosphorylation and inhibitory phosphorylation of glycogen synthase kinase-3β (GSK-3β) at Ser9, consistent with increased GSK-3β activity. This was accompanied by decreased accumulation of transcriptionally active β-catenin, impaired nuclear β-catenin localization, and suppression of canonical Wnt target genes (Axin2, Lef1, Ccnd1, c-Myc). Pharmacological inhibition of GSK-3β partially restored β-catenin signaling and osteogenic differentiation, whereas inhibition of Toll-like receptor 4 (TLR4) attenuated heme-induced suppression of the AKT/GSK-3β pathway. Combined inhibition of TLR4 and GSK-3β produced the most pronounced restoration of osteogenesis in the presence of heme. Consistent with the in vitro findings, maternal heme administration during pregnancy significantly reduced ALP activity in multiple fetal craniofacial ossification centers in vivo. Collectively, these findings demonstrate that extracellular heme suppresses osteogenesis and supports a model in which TLR4-associated dysregulation of the AKT/GSK-3β/β-catenin axis contributes to impaired osteogenic differentiation.

## 1. Introduction

Bone formation is a tightly regulated process that depends on the differentiation of osteoprogenitor cells into mature osteoblasts capable of producing and mineralizing the extracellular matrix [1,2]. Proper osteoblast differentiation is essential for skeletal development, maintenance of bone mass, and regeneration following injury [3,4]. Impaired osteogenesis contributes to a broad range of skeletal disorders, including osteoporosis, delayed bone healing, craniofacial abnormalities, and inflammatory bone diseases [1,2]. Understanding the molecular factors that regulate osteoblast differentiation therefore remains a major objective in skeletal biology.

Bone regeneration occurs within a highly dynamic inflammatory microenvironment. Following skeletal injury, disruption of local vasculature leads to formation of a fracture hematoma that serves as a transient niche for inflammatory cells, mesenchymal progenitors, and morphogenetic signals that initiate tissue repair [5,6]. Although early inflammatory responses are required for successful regeneration, excessive or persistent inflammatory signaling can impair osteoblast differentiation and compromise bone formation. Increasing evidence indicates that interactions between the skeletal and immune systems, collectively referred to as osteoimmunology [7,8], play critical roles in determining the balance between bone formation and bone resorption [9,10,11,12]. While considerable attention has focused on cytokines and pathogen-associated signals, much less is known about the influence of endogenous danger-associated molecular patterns (DAMPs) released during hemorrhage and tissue injury on osteoblast differentiation and bone formation.

Extracellular heme is a candidate mediator linking hemorrhage-associated inflammation to impaired osteogenesis. Heme is an iron-containing prosthetic group that is essential for numerous physiological processes, including oxygen transport, mitochondrial respiration, and cellular redox regulation [13]. Under pathological conditions such as hemolysis, hemorrhage, ischemia–reperfusion injury, trauma, and severe inflammation, extracellular heme accumulates when endogenous scavenging systems become saturated [13,14,15]. In this extracellular setting, heme functions as a potent DAMP that promotes inflammation, oxidative stress, vascular dysfunction, and tissue injury through activation of innate immune signaling pathways [9,11,16,17]. Extracellular heme has been shown to activate TLR4 signaling in endothelial cells, leading to NF-κB activation and endothelial responses [12]. Elevated extracellular heme has been implicated in the pathogenesis of diverse disorders, including sickle cell disease, hemolytic anemia, sepsis, and vascular inflammatory diseases [12,18,19]. Although heme has been reported to affect osteoblast differentiation and mineralization, the signaling mechanisms underlying these effects remain incompletely understood.

The potential relevance of extracellular heme to skeletal biology is supported by observations linking hemolytic and inflammatory conditions with impaired bone health. Patients with chronic hemolytic disorders frequently exhibit reduced bone mass, skeletal abnormalities, and an increased risk of fractures [20,21]. Moreover, bone repair occurs within inflammatory and hemorrhagic microenvironments in which erythrocyte degradation and local release of heme are unavoidable consequences of vascular disruption [22,23,24]. These findings raise the possibility that extracellular heme directly influences osteogenic cell behavior and contributes to impaired bone formation in hemolytic and inflammatory conditions. However, whether TLR4/AKT/GSK-3β/β-catenin signaling contributes to the effects of extracellular heme exposure on osteoblast differentiation remains unclear.

Among the pathways governing osteoblast differentiation, canonical Wnt/β-catenin signaling is a master regulator of osteogenesis [3,25]. Activation of β-catenin promotes osteoblast commitment, maturation, and matrix mineralization, whereas suppression of this pathway profoundly impairs bone formation [26,27]. β-catenin stability is tightly regulated by glycogen synthase kinase-3β (GSK-3β), which targets β-catenin for degradation. In turn, AKT signaling enhances β-catenin activity through inhibitory phosphorylation of GSK-3β [26]. Dysregulation of the AKT/GSK-3β/β-catenin signaling axis has emerged as an important mechanism underlying impaired osteogenesis in inflammatory microenvironments, making this pathway a compelling candidate through which extracellular heme may influence osteoblast differentiation.

Toll-like receptor 4 (TLR4) may provide an important mechanistic link between extracellular heme and osteogenic dysfunction [12,28]. Extracellular heme has been shown to activate TLR4-dependent signaling pathways in multiple cell types [9,28]; Belcher et al. further demonstrated that heme-induced responses were inhibited by TAK-242 and were absent in TLR4-deficient endothelial cells [12]. Growing evidence indicates that TLR4 signaling modulates osteoblast differentiation and skeletal remodeling [29,30]. These observations suggest that heme-induced activation of innate immune signaling may directly intersect with pathways governing osteogenesis.

In the present study, we investigated the effects of heme on osteogenesis and explored the underlying molecular mechanisms. Using an in vitro model of osteoblast differentiation, we demonstrate that heme suppresses osteogenesis and impairs canonical Wnt/β-catenin signaling. These findings were further supported by an in vivo model of craniofacial bone development. Mechanistically, our findings support a model in which TLR4-associated dysregulation of the AKT/GSK-3β/β-catenin axis contributes to heme-induced impairment of osteogenic differentiation. These findings extend previous observations on the effects of heme in osteoblasts and provide a mechanistic framework linking extracellular heme-associated danger signaling to impaired bone formation in inflammatory and hemorrhagic microenvironments.

## 2. Results

### 2.1. Extracellular Heme Inhibits Osteoblast Differentiation, Matrix Mineralization and Canonical Wnt/β-Catenin Signaling

**Figure 1:**
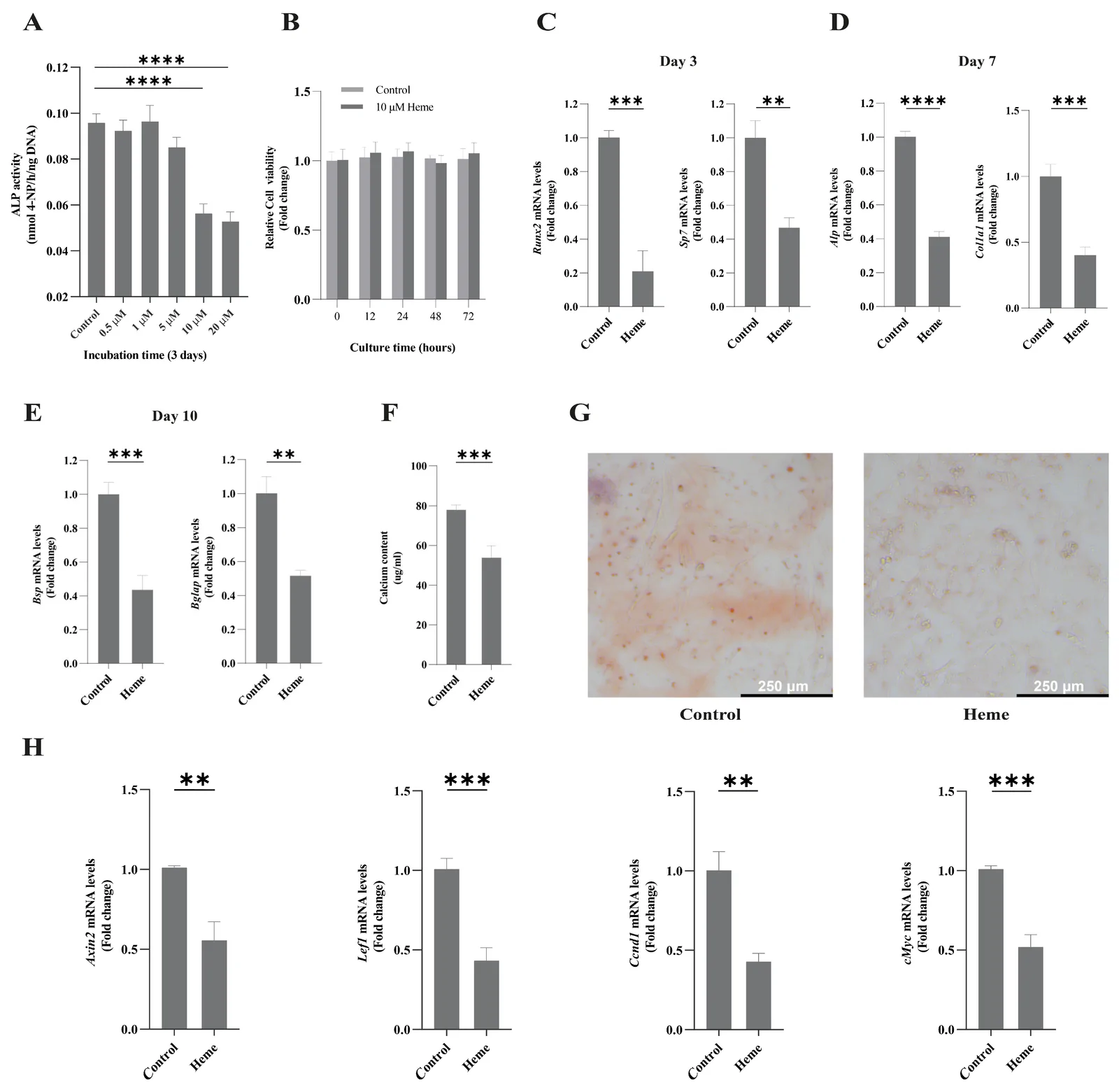
Extracellular heme inhibits osteoblast differentiation, matrix mineralization and Wnt/β-catenin signaling target gene expression. MC3T3-E1 cells were cultured under osteogenic conditions. (**A**) Cells were treated with the indicated concentrations of heme for 3 days, after which ALP activity was quantified. (**B**) Cell viability after treatment with 10 μM heme for 12–72 h was assessed using the CCK-8 assay; 10 μM heme was used in subsequent experiments. (**C**–**E**) RT-qPCR analysis of osteogenic differentiation markers: Runx2 and Sp7 at day 3, Alpl and Col1a1 at day 7, and Bsp and Bglap at day 10. (**F**) Quantification of calcium deposition at day 14. (**G**) Representative Alizarin Red S staining images showing matrix mineralization at day 21. (**H**) RT-qPCR analysis of canonical Wnt target genes (Axin2, Lef1, Ccnd1, and c-Myc) following 24 h of heme treatment. Data are presented as mean ± SD from three independent experiments. Statistical comparisons are indicated in the graphs. ** *p* < 0.01, *** *p* < 0.001, **** *p* < 0.0001.

To determine whether extracellular heme affects osteogenic differentiation, MC3T3-E1 pre-osteoblasts were cultured under osteogenic conditions in the presence of increasing concentrations of heme. Heme significantly reduced ALP activity in a dose-dependent manner at day 3, with pronounced inhibition observed at concentrations of 10–20 μM (Figure 1A). Importantly, treatment with 10 μM heme did not affect cell viability at any of the examined time points (0, 12, 24, 48, and 72 h) (Figure 1B), indicating that the observed reduction in osteogenic differentiation was not attributable to cytotoxicity. This concentration was therefore selected for subsequent experiments.

Limulus amebocyte lysate (LAL) analysis showed that the absorbance of 20 μM heme was below that of the lowest endotoxin standard tested (0.010 EU/mL) (Supplementary Figure S1).

To investigate the impact of heme throughout the osteogenic program, expression of osteogenic markers was assessed by RT-qPCR. Heme significantly reduced expression of the osteogenic transcription factors Runx2 and Sp7 at day 3 (Figure 1C), indicating impaired osteogenic commitment. Furthermore, expression of the differentiation markers Alpl and Col1a1 was markedly suppressed at day 7 (Figure 1D), while the osteogenic markers Bsp and Bglap were significantly reduced at day 10 (Figure 1E).

Consistent with these transcriptional changes, heme substantially decreased extracellular matrix mineralization. Quantitative calcium measurements revealed reduced calcium deposition at day 14 (Figure 1F), whereas Alizarin Red S staining demonstrated markedly impaired mineralized nodule formation at day 21 (Figure 1G).

Because canonical Wnt/β-catenin signaling is a central regulator of osteoblast differentiation, we next examined whether heme affects this pathway. To evaluate the transcriptional consequences of β-catenin, expression of canonical Wnt target genes was examined. Heme significantly reduced expression of Axin2, Lef1, Ccnd1, and c-Myc (Figure 1H), indicating impaired β-catenin-dependent transcriptional activity.

Collectively, these findings indicate that heme downregulates Wnt/β-catenin target genes, suppresses osteoblast differentiation across multiple stages of osteogenesis, and ultimately impairs matrix mineralization.

### 2.2. Pharmacological Inhibition of GSK-3β Partially Restores Wnt/β-Catenin Signaling and Osteogenic Differentiation

Canonical Wnt/β-catenin signaling is thus involved in heme-induced suppression of osteogenic differentiation. To examine whether increased GSK-3β activity contributes functionally to the anti-osteogenic effects of heme, cells were treated with the GSK-3β inhibitor LiCl (1 mM) in the presence of heme.

**Figure 2:**
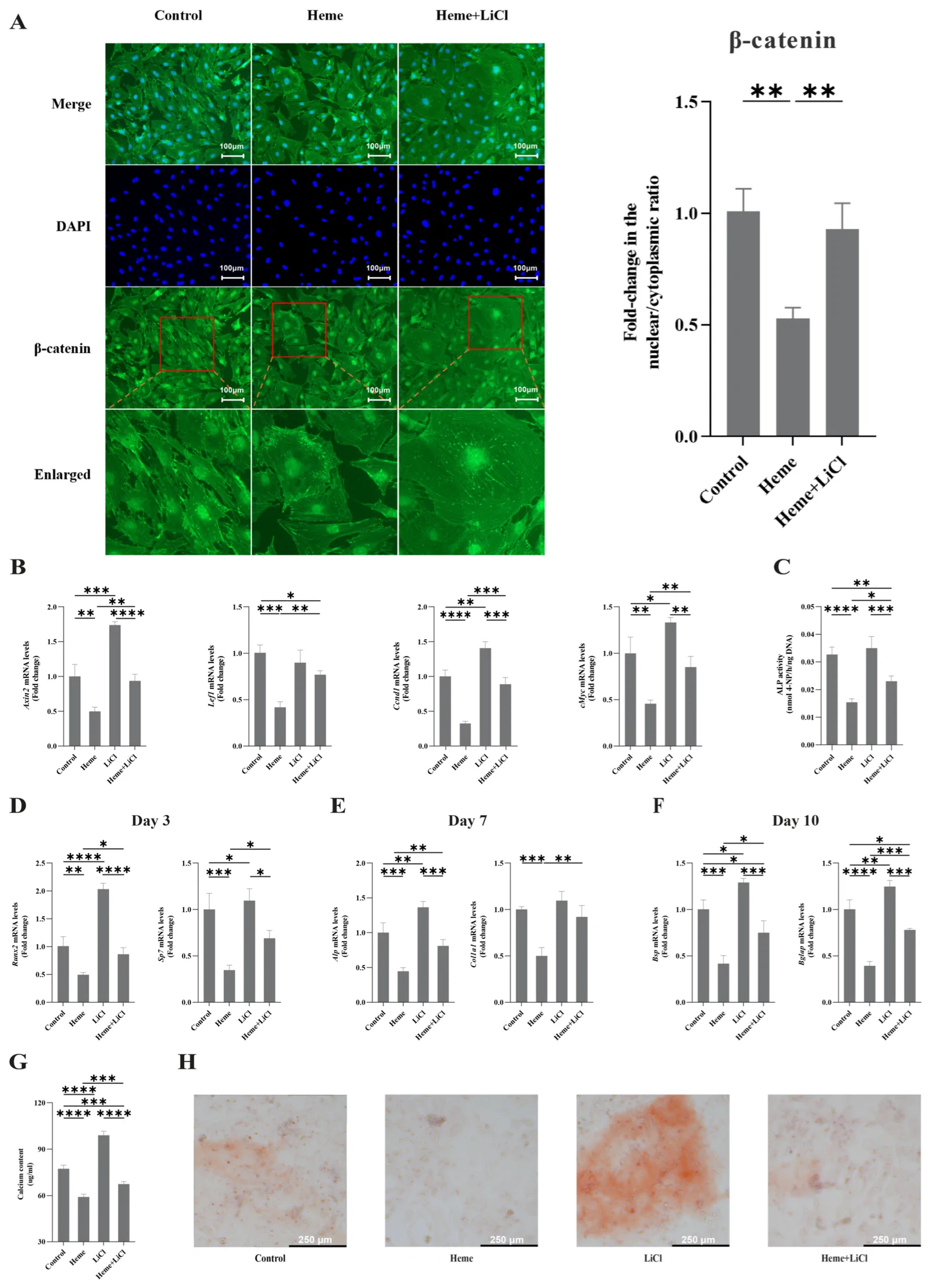
GSK-3β inhibition partially restores β-catenin signaling and osteogenic differentiation in heme-treated cells. MC3T3-E1 cells were treated with heme (10 μM), LiCl (1 mM), or both. (**A**) Representative immunofluorescence images of β-catenin localization and quantification of the nuclear-to-cytoplasmic fluorescence intensity ratio after 6 h of treatment. β-Catenin is shown in green and nuclei are counterstained with DAPI (blue); red boxes indicate the regions shown at higher magnification. Scale bar = 100 μm. (**B**) Expression of β-catenin target genes (Axin2, Lef1, Ccnd1, and c-Myc) after 24 h was determined by RT-qPCR. (**C**) ALP activity measured at day 3. (**D**–**F**) Expression of the osteogenic markers Runx2 and Sp7 at day 3, Alpl and Col1a1 at day 7, and Bsp and Bglap at day 10. (**G**) Calcium deposition was measured at day 14. (**H**) Representative Alizarin Red S staining images showing matrix mineralization at day 21. Data are presented as mean ± SD from three independent experiments. Statistical comparisons are indicated in the graphs. * *p* < 0.05, ** *p* < 0.01, *** *p* < 0.001, **** *p* < 0.0001.

Immunofluorescence analysis showed diminished nuclear accumulation of β-catenin after 6 h of heme exposure (Figure 2A), demonstrating impaired β-catenin nuclear translocation. This observation indicates that extracellular heme suppresses canonical Wnt/β-catenin signaling.

In heme-exposed cells, LiCl partially restored nuclear accumulation of β-catenin (Figure 2A). Consistent with this finding, LiCl partially rescued the heme-induced suppression of the β-catenin target genes Axin2, Lef1, Ccnd1, and c-Myc after 24 h, while also increasing their expression when administered alone (Figure 2B).

This partial restoration of β-catenin signaling was accompanied by a marked recovery of osteogenic differentiation. Specifically, LiCl partially reversed the inhibitory effects of heme on ALP activity (Figure 2C); the expression of osteogenic markers Runx2, Sp7, Alpl, Col1a1, Bsp and Bglap (Figure 2D–F); as well as calcium deposition and matrix mineralization (Figure 2G,H). LiCl alone also promoted these osteogenic responses.

Although the rescue was incomplete, these results demonstrate that activation of GSK-3β contributes substantially to heme-induced suppression of osteogenesis.

### 2.3. Simultaneous Inhibition of TLR4 and GSK-3β Markedly Restores Wnt/β-Catenin Signaling and Osteogenic Differentiation

The incomplete rescue of osteogenesis by LiCl suggested that additional pathways may contribute to the inhibitory effects of heme. Because extracellular heme is a well-established endogenous ligand of TLR4, we therefore investigated whether TLR4 signaling contributes to heme-induced suppression of osteogenic differentiation. To further determine whether simultaneous targeting of TLR4 signaling and GSK-3β activity could improve osteogenic recovery, cells were treated with heme, LiCl, or their combination, with the TLR4 inhibitor TAK-242 (1 μM) administered 2 h before heme exposure where indicated.

**Figure 3:**
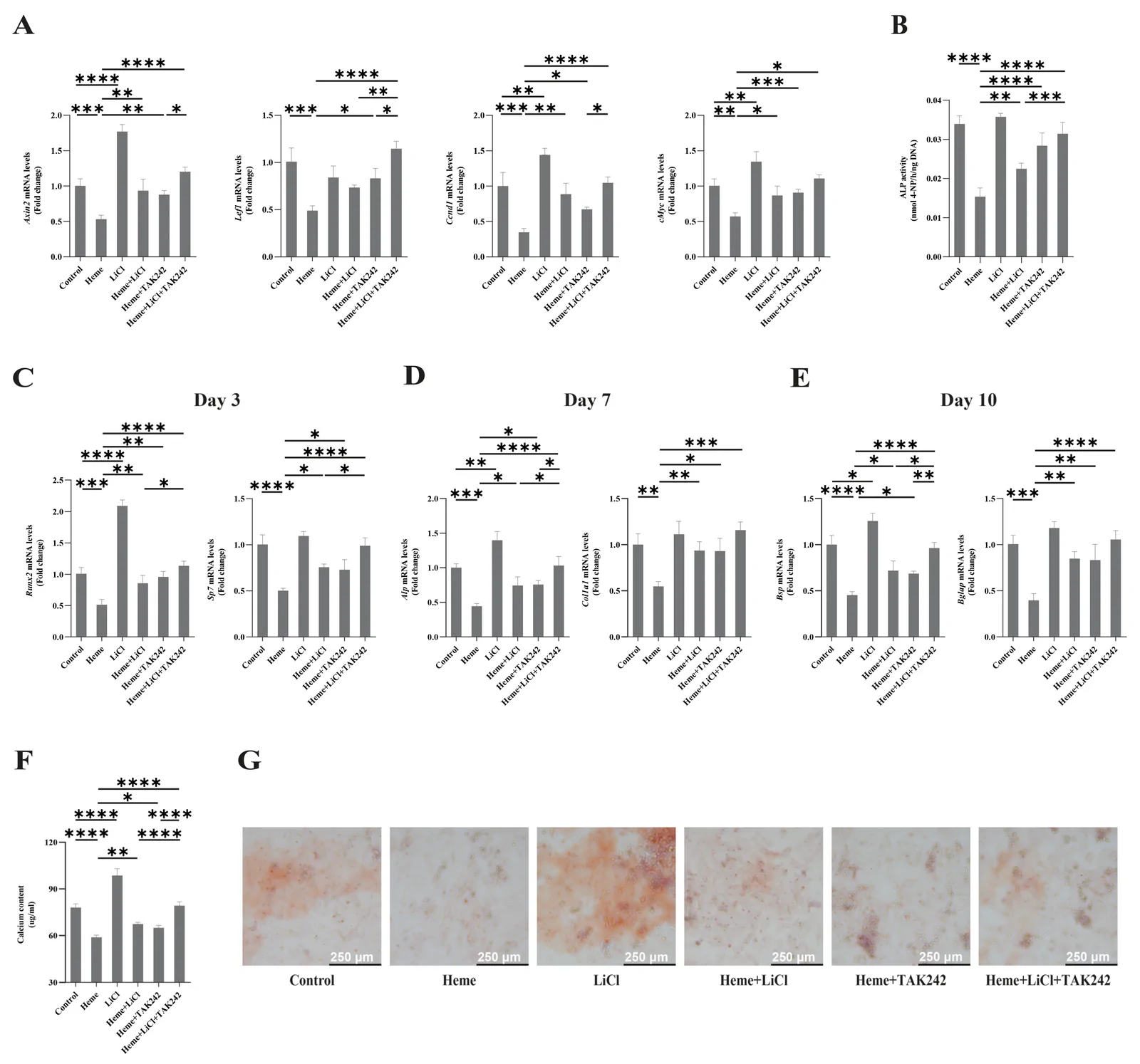
Combined inhibition of TLR4 and GSK-3β effectively restores β-catenin activity and osteogenic mineralization. MC3T3-E1 cells were treated with heme (10 μM), TAK-242 (1 μM), LiCl (1 mM), or the indicated combinations. TAK-242 (1 μM) was administered 2 h before heme exposure. (**A**) Expression of β-catenin target genes (Axin2, Lef1, Ccnd1, and c-Myc) after 24 h determined by RT-qPCR. (**B**) ALP activity measured at day 3. (**C**–**E**) Expression the osteogenic markers of Runx2 and Sp7 at day 3, Alpl and Col1a1 at day 7, and Bsp and Bglap at day 10. (**F**) Calcium deposition was measured at day 14. (**G**) Representative Alizarin Red S staining showing extracellular matrix mineralization at day 21. Data are presented as mean ± SD from three independent experiments. Statistical comparisons are indicated in the graphs. * *p* < 0.05, ** *p* < 0.01, *** *p* < 0.001, **** *p* < 0.0001.

LiCl alone increased the expression of Wnt/β-catenin target genes, whereas LiCl or TAK-242 partially restored their suppression in heme-exposed cells. Notably, combined treatment produced a greater recovery, with expression levels approaching those of untreated controls (Figure 3A).

This rescue of Wnt/β-catenin signaling translated into substantial recovery of osteogenic function. LiCl alone also promoted osteogenic differentiation. In heme-exposed cells, LiCl or TAK-242 partially reversed the inhibitory effects of heme on ALP activity (Figure 3B) and osteogenic marker expression (Figure 3C–E), whereas combined treatment produced a more pronounced recovery, restoring ALP activity and osteogenic marker expression to near-control levels (Figure 3B–E). The combined treatment also markedly improved calcium deposition and matrix mineralization (Figure 3F,G).

### 2.4. TLR4 Contributes to Heme-Induced Suppression of the AKT/GSK-3β/β-Catenin Axis

To investigate the mechanism underlying heme-induced suppression of β-catenin signaling, we examined the AKT/GSK-3β pathway, a critical regulator of β-catenin stability during osteogenesis.

**Figure 4:**
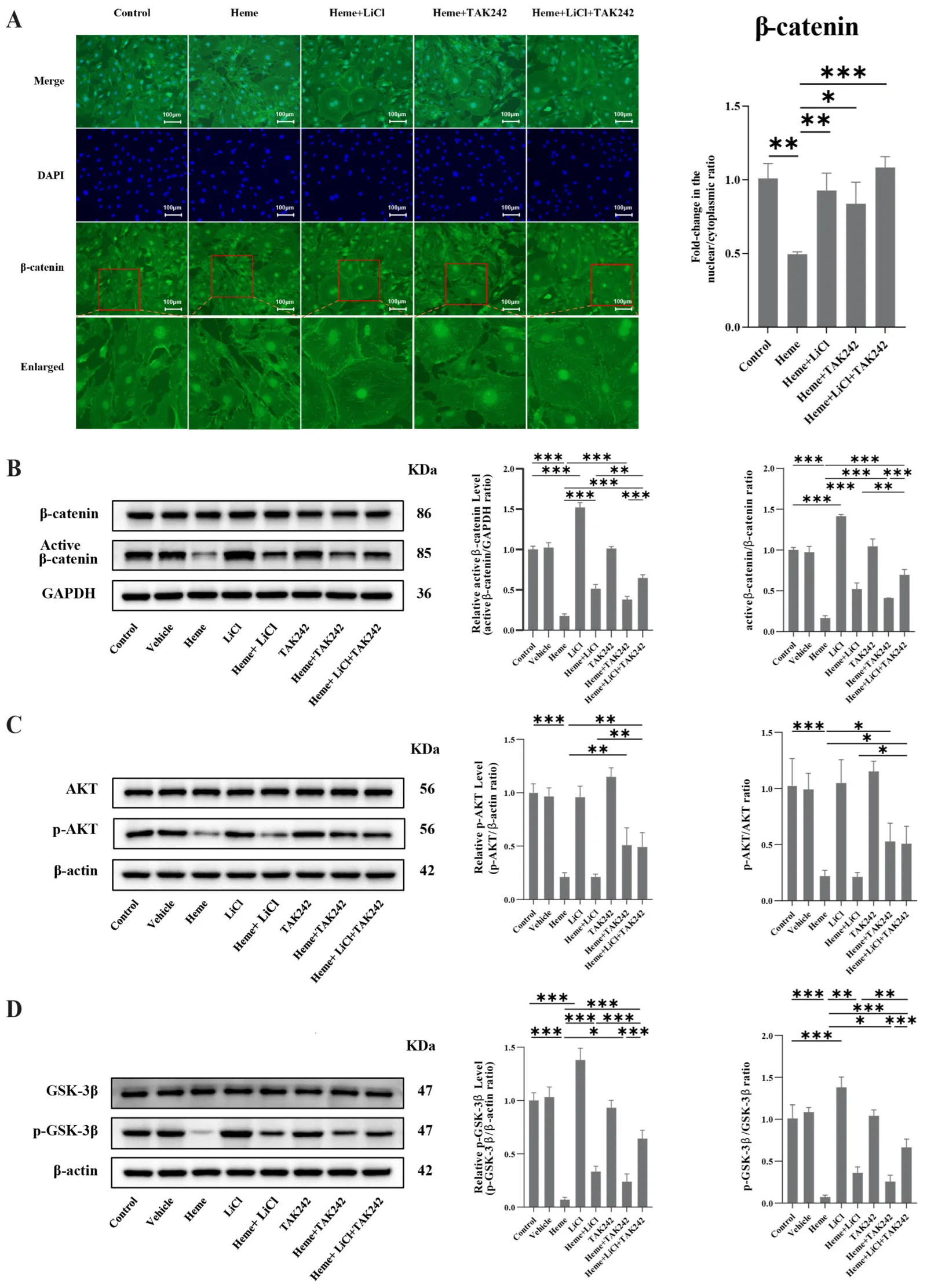
TLR4 contributes to heme-mediated suppression of the AKT/GSK-3β signaling axis. MC3T3-E1 cells were treated with heme (10 μM), LiCl (1 mM), and/or TAK-242 (1 μM), as indicated. For TAK-242-containing groups, cells were pretreated with TAK-242 for 2 h before heme exposure. (**A**) Representative immunofluorescence images of β-catenin localization and quantification of the nuclear-to-cytoplasmic fluorescence intensity ratio after 6 h of treatment. β-Catenin is shown in green and nuclei are counterstained with DAPI (blue); red boxes indicate the regions shown at higher magnification. Scale bar = 100 μm. (**B**) Representative immunoblots and densitometric analysis of active (non-phosphorylated) β-catenin and total β-catenin after 24 h of treatment. Active β-catenin levels were normalized to GAPDH and expressed relative to control, and the active β-catenin/β-catenin ratio was also calculated. (**C**) Representative Western blots and densitometric quantification of phosphorylated AKT (Ser473) and total AKT after 24 h. (**D**) Representative Western blots and quantification of phosphorylated GSK-3β (Ser9) and total GSK-3β after 24 h. Data are presented as mean ± SD from three independent experiments. Statistical comparisons are shown in the graphs. * *p* < 0.05, ** *p* < 0.01, *** *p* < 0.001.

Immunofluorescence showed markedly reduced nuclear accumulation of β-catenin in heme-treated cells. In heme-exposed cells, either LiCl treatment or pretreatment with TAK-242 partially restored β-catenin nuclear accumulation, whereas combined treatment restored nuclear β-catenin to levels comparable to those in untreated controls (Figure 4A).

We next examined β-catenin activation at the protein level. Western blot analysis demonstrated that heme significantly reduced levels of transcriptionally active, non-phosphorylated β-catenin (dephospho-Ser37/Thr41) after 24 h of exposure, whereas total β-catenin protein levels remained largely unchanged (Figure 4B). These findings suggest that heme primarily suppresses β-catenin activation rather than overall protein abundance. LiCl treatment alone significantly increased active β-catenin levels, whereas TAK-242 alone had little effect. In heme-exposed cells, either LiCl or TAK-242 partially restored active β-catenin, while their combination produced a greater recovery than either treatment alone (Figure 4B).

To further define the signaling events upstream of β-catenin, we next assessed AKT activation. Heme significantly reduced AKT phosphorylation at Ser473 without affecting total AKT protein levels (Figure 4C), indicating decreased AKT activation. TAK-242 partially restored AKT phosphorylation in heme-exposed cells (Figure 4C), whereas LiCl did not appreciably reverse the heme-induced reduction in p-AKT. Moreover, combined treatment with TAK-242 and LiCl did not further increase AKT phosphorylation beyond that achieved with TAK-242 alone (Figure 4C). These findings suggest that the effect of TLR4 inhibition occurs at or upstream of AKT, whereas the action of LiCl is primarily exerted downstream of AKT.

Because activated AKT negatively regulates GSK-3β through inhibitory phosphorylation at Ser9, we next assessed GSK-3β phosphorylation. Heme significantly decreased Ser9 phosphorylation of GSK-3β while total GSK-3β expression remained unchanged (Figure 4D). Reduced inhibitory phosphorylation is consistent with increased GSK-3β activity, which is expected to suppress β-catenin signaling. This provides a mechanistic basis for the observed reduction in active and nuclear β-catenin. LiCl alone markedly increased phosphorylation of GSK-3β, whereas TAK-242 alone had little effect. In heme-exposed cells, either LiCl or TAK-242 partially rescued GSK-3β Ser9 phosphorylation, while combined treatment produced a substantially greater recovery than either intervention alone (Figure 4D).

Together, these findings support a model in which TLR4 signaling contributes to heme-induced suppression of AKT activation, leading to reduced inhibitory phosphorylation of GSK-3β and subsequent impairment of β-catenin activation and nuclear accumulation. The enhanced recovery of GSK-3β phosphorylation and β-catenin signaling after combined TAK-242 and LiCl treatment further supports the involvement of both TLR4-associated signaling and GSK-3β regulation in heme-mediated suppression of osteogenic differentiation.

### 2.5. Maternal Heme Exposure Suppresses Osteogenic Activity During Craniofacial Bone Development

To determine whether the anti-osteogenic effects of heme observed in vitro are reflected in vivo, osteogenic activity was evaluated in fetal craniofacial tissues following maternal heme administration.

**Figure 5:**
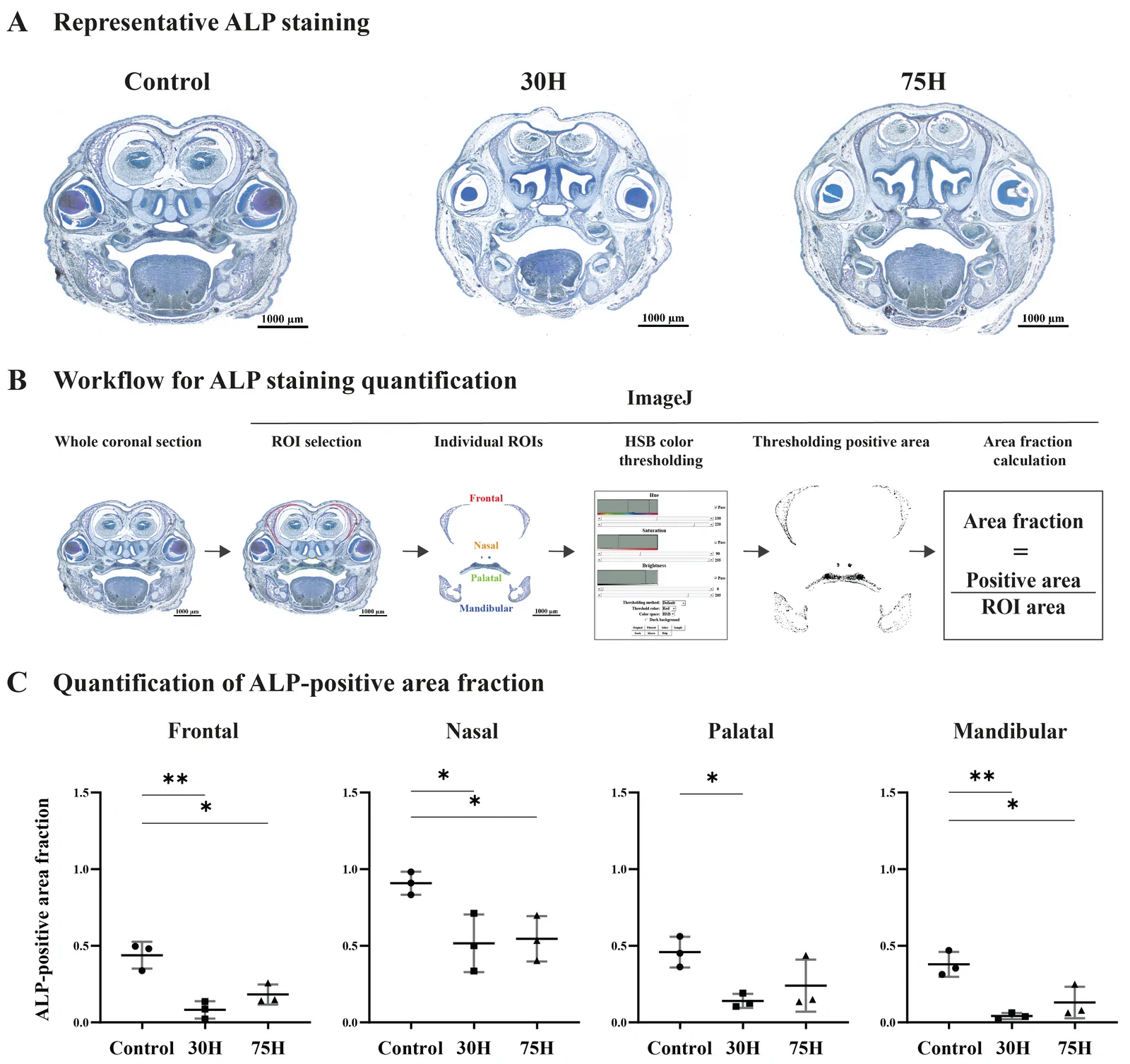
Maternal heme exposure reduces osteogenic activity during fetal craniofacial bone development. (**A**) Representative ALP staining of coronal craniofacial sections from E16 fetuses derived from control dams or dams treated with 30 or 75 μmol/kg heme (30H and 75H, respectively). (**B**) Schematic overview of the image analysis workflow used to quantify ALP-positive areas in the frontal, nasal, palatal and mandibular regions. Colors indicate the regions of interest: frontal (red), nasal (orange), palatal (green), and mandibular (blue). (**C**) Quantification of ALP-positive area fractions within individual craniofacial ossification centers. Data are presented as mean ± SD (*n* = 3 embryos per group). Statistical comparisons are indicated in the graphs. * *p* < 0.05, ** *p* < 0.01.

ALP staining of embryonic day 16 (E16) fetal craniofacial sections revealed a marked reduction in osteogenic activity in heme-exposed embryos compared with controls (Figure 5A). To quantify these changes across distinct craniofacial ossification centers, ALP-positive areas were analyzed in the frontal, nasal, palatal and mandibular regions using the image analysis workflow shown in Figure 5B.

Quantitative analysis demonstrated significantly reduced ALP-positive area fractions in the frontal, nasal and mandibular regions following exposure to either 30 or 75 μmol/kg heme compared with controls (Figure 5C). In the palatal region, the ALP-positive area fraction was significantly reduced in the 30 μmol/kg heme group, whereas the reduction observed in the 75 μmol/kg group did not reach statistical significance (Figure 5C). Thus, reduced ALP activity was observed across multiple anatomically distinct craniofacial ossification centers following maternal heme exposure.

The broad reduction in ALP activity across several craniofacial skeletal regions indicates that elevated extracellular heme suppresses osteogenic activity in vivo during periods of active bone formation. These findings corroborate the in vitro data and support the concept that extracellular heme acts as a negative regulator of osteogenesis.

## 3. Discussion

The present study demonstrates that extracellular heme negatively regulates osteogenesis and identifies a signaling mechanism underlying this effect. We demonstrate that heme suppresses osteoblast differentiation, inhibits matrix mineralization, attenuates canonical Wnt/β-catenin signaling in vitro, and reduces osteogenic activity during craniofacial skeletal development in vivo. Mechanistically, our findings support a model in which extracellular heme is consistent with TLR4-dependent signaling, suppresses AKT activity, enhances GSK-3β activity, reduces β-catenin signaling, and ultimately impairs osteogenic gene expression and mineralized matrix formation. These findings establish a mechanistic link between hemolysis-associated danger signaling and impaired bone formation and identify TLR4-associated dysregulation of the AKT/GSK-3β/β-catenin axis as a mechanism contributing to heme-induced impairment of osteogenesis.

A major finding of this study is that extracellular heme directly suppresses osteoblast differentiation. Previous studies have also examined the effects of hemin on osteoblast differentiation and mineralization. Lin et al. used hemin as an inducer of HO-1 in primary osteoblasts and showed that hemin increased HO-1 expression and decreased ALP, osteocalcin, and RUNX2 mRNA expression, as well as calcium deposition during osteogenic culture [31]. Lee et al. showed that hemin treatment reduced ALP activity, calcium accumulation, bone-like nodule formation, and Runx2 and osteocalcin expression in MC3T3-E1 cells, while increasing HO-1 expression [32]. In that study, hemin was used as a chemical inducer of HO-1 [32]. More recently, Soto et al. reported that low concentrations of hemin increased proliferation and migration of MC3T3-E1 pre-osteoblasts, did not change osteoblastic differentiation after 7 days, but postponed mineralization after 14 days of culture [33]. Our study extends these previous observations by examining the association of heme exposure with TLR4/AKT/GSK-3β/β-catenin signaling during osteogenic differentiation. The biological relevance of this observation may extend beyond classical hemolytic disorders.

Bone regeneration occurs within a fracture hematoma, a highly specialized immune-regulated microenvironment that coordinates inflammatory, angiogenic, and osteogenic processes required for successful healing [14,15,34]. Local release of extracellular heme is therefore an unavoidable consequence of skeletal trauma. Our findings suggest that excessive extracellular heme exposure may become maladaptive by directly suppressing osteoblast differentiation and mineralized matrix formation, thereby influencing regenerative outcome.

Importantly, these observations should not be interpreted to suggest that hematoma formation is detrimental per se. Formation of a fracture hematoma is essential for normal skeletal repair, providing inflammatory, angiogenic, and osteogenic cues required for successful regeneration [34]. Our findings instead suggest that excessive accumulation or prolonged persistence of extracellular heme may shift this otherwise beneficial microenvironment toward a state that is less permissive for osteoblast differentiation. Future studies should determine whether extracellular heme exerts concentration- and time-dependent effects during different phases of tissue repair. The implications of these findings may also be relevant to conditions associated with chronic or recurrent hemolysis. Patients with sickle cell disease and β-thalassemia frequently exhibit reduced bone mineral density, skeletal abnormalities, delayed bone repair, and increased fracture risk [20,21,22,23,35]. Although these complications are generally attributed to chronic inflammation, marrow expansion, endocrine dysfunction, or altered mineral metabolism, our findings raise the possibility that extracellular heme itself contributes directly to impaired skeletal homeostasis by suppressing osteoblast differentiation. Although the present study does not establish causality in these diseases, it provides a mechanistic framework through which persistent exposure to hemolysis-derived danger signals could negatively influence bone formation. Elevated local free heme has been reported in periodontitis [36]. In gingival crevicular fluid, free heme concentrations were significantly higher at periodontally diseased sites than at healthy sites and were positively correlated with probing pocket depth, attachment level, and radiographic bone loss [36]. A subsequent study likewise reported higher gingival crevicular fluid free heme levels at periodontitis sites than at healthy sites in both smokers and nonsmokers [37]. In addition, the lysine-specific gingipain K of Porphyromonas gingivalis can cleave hemoglobin, haptoglobin, and hemopexin, which may provide the bacterium with a usable source of heme for growth [38]. Other bacteria may also increase local heme availability through distinct mechanisms. Prevotella intermedia expresses the cysteine protease interpain A, which promotes hemoglobin oxidation and, under acidic conditions, hemoglobin degradation with heme release [39]. Treponema denticola can lyse human erythrocytes, an activity proposed to favor higher heme concentrations in periodontal sites [40]. Beyond periodontal bacteria, Streptococcus pneumoniae releases hemoglobin through pneumolysin-mediated hemolysis, while bacterial hydrogen peroxide promotes hemoglobin oxidation and subsequent heme release and degradation [41].

More broadly, these observations extend current concepts in osteoimmunology by linking hemolysis-associated danger signaling to the regulation of skeletal regeneration. Most studies in osteoimmunology have focused on cytokines, microbial ligands, or immune-cell-derived mediators as regulators of bone formation. In contrast, the present findings identify extracellular heme, a hemolysis-derived danger-associated molecular pattern, as a direct regulator of osteoblast differentiation. This expands current osteoimmunological concepts by suggesting that endogenous products generated during hemorrhage and erythrocyte breakdown can directly influence skeletal cell fate decisions.

Another key finding is that heme exposure targets canonical Wnt/β-catenin signaling. Canonical Wnt signaling is indispensable for osteoblast commitment, maturation, and skeletal development [3,4,26,27,42,43,44].

Consistent with this central role, heme reduced levels of active β-catenin, diminished its nuclear accumulation, and suppressed expression of multiple canonical Wnt target genes. These effects were accompanied by reduced expression of osteogenic differentiation markers, indicating broad suppression of the osteogenic program. To our knowledge, this is the first study to show that extracellular heme suppresses canonical Wnt/β-catenin signaling in osteogenic cells. Interestingly, suppression of Wnt/β-catenin signaling has emerged as a common consequence of inflammatory and stress-associated signaling pathways in skeletal tissues, suggesting that heme may converge on regulatory mechanisms shared with other inflammatory mediators.

**Figure 6:**
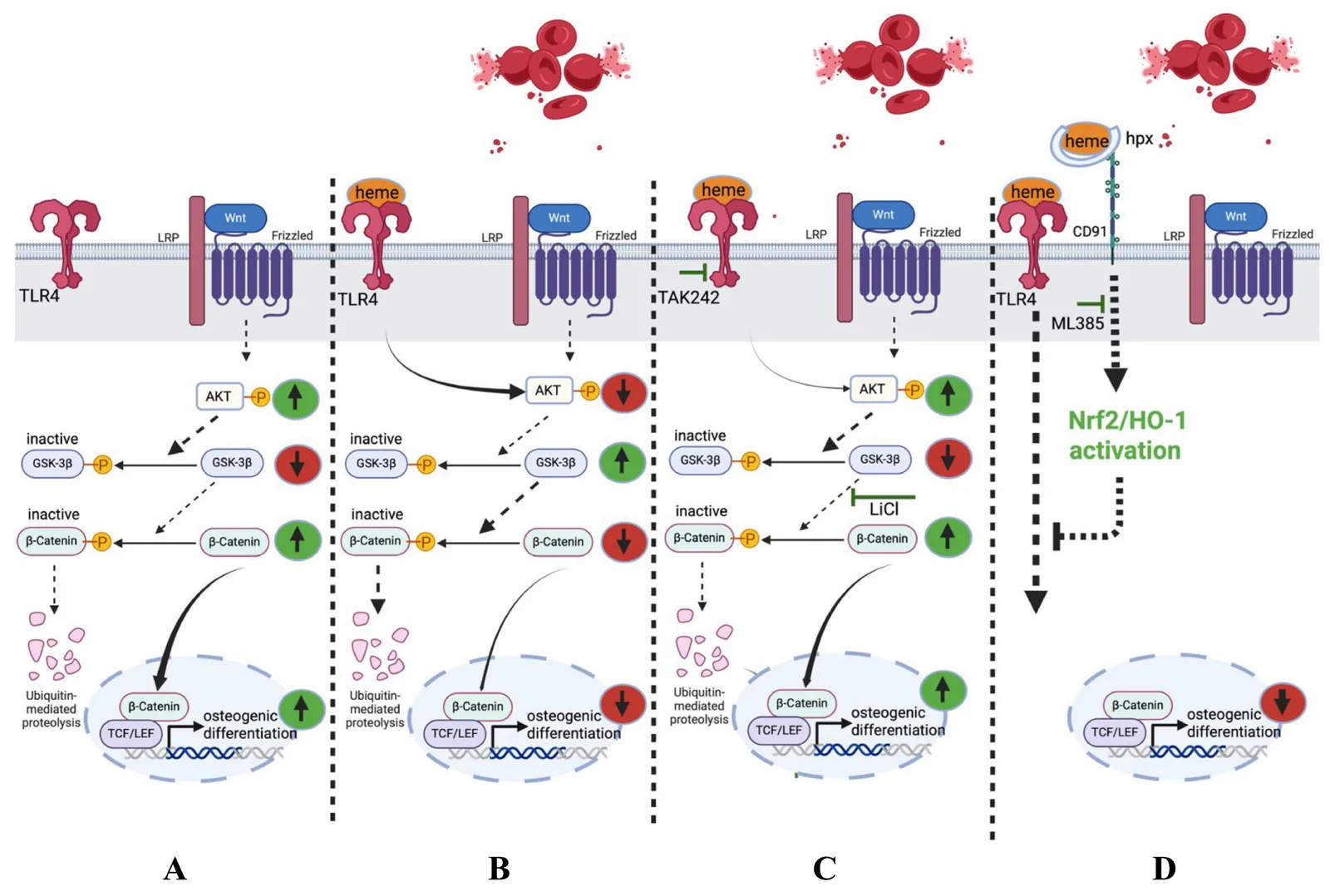
Proposed mechanism by which heme suppresses osteogenic differentiation. (**A**) Under physiological osteogenic conditions, AKT phosphorylates GSK-3β at Ser9, thereby inhibiting its activity. This promotes β-catenin stabilization, nuclear translocation, and transcriptional activity of canonical Wnt target genes, leading to osteoblast differentiation and matrix mineralization. (**B**) Heme activates TLR4 signaling, resulting in reduced AKT activation and decreased inhibitory phosphorylation of GSK-3β. Enhanced GSK-3β activity promotes β-catenin degradation, suppresses canonical Wnt signaling, and inhibits osteogenic gene expression, osteoblast differentiation, and matrix mineralization. (**C**) Pharmacological inhibition of TLR4 (TAK-242) and GSK-3β (LiCl) restores AKT/GSK-3β/β-catenin signaling, rescues canonical Wnt activity, and reverses the inhibitory effects of heme on osteogenic differentiation and mineralized matrix formation. However, additional heme-responsive pathways may also contribute. (**D**) Exogenous heme scavenged by hemopexin (Hpx) may be internalized through the CD91/LRP1 receptor complex and promote activation of cytoprotective Nrf2/HO-1 signaling. Pharmacological inhibition of Nrf2 with ML385 further exacerbated heme-induced suppression of osteogenic gene expression, suggesting that endogenous Nrf2 activity partially counteracts the anti-osteogenic effects of extracellular heme and forms a putative protective pathway. Green upward arrows indicate increases, red downward arrows indicate decreases, green T-shaped lines indicate pharmacological inhibition, and yellow “P” symbols indicate phosphorylation. Connecting arrows indicate pathway relationships; the dashed black T-shaped line in panel (**D**) indicates the proposed protective effect of Nrf2/HO-1 signaling. Created with Biorender.com.

Our findings further identify the AKT/GSK-3β pathway as a critical signaling node linking extracellular heme to impaired osteogenesis. Extracellular heme has been shown to activate TLR4 signaling [12,16]. TLR4-mediated PI3K/AKT/GSK-3β signaling has been reported in rat hepatocytes [45], while PI3K/AKT/GSK3 signaling has been implicated in the regulation of osteogenesis (Figure 6A) [46]. Consistent with this framework, heme reduced AKT activation and inhibitory phosphorylation of GSK-3β, providing a plausible explanation for the observed suppression of β-catenin signaling (Figure 6B) [12,16,45,46,47]. Pharmacological inhibition of GSK-3β partially restored β-catenin activity, osteogenic gene expression, and matrix mineralization. However, the incomplete rescue suggests that heme engages additional pathways that contribute to osteogenic dysfunction. Given the pleiotropic biological effects of extracellular heme, future transcriptomic and phosphoproteomic studies may help define the broader signaling network involved.

An additional mechanistic insight provided by this study is the involvement of TLR4 signaling. Previous studies have established that extracellular heme can activate TLR4 signaling. In endothelial cells, Belcher et al. demonstrated that heme-induced responses were inhibited by TAK-242 and were absent in TLR4-deficient cells, providing strong evidence for TLR4-mediated heme signaling [12]. Here, pharmacological inhibition of TLR4 partially reversed the suppressive effects of heme on AKT/GSK-3β signaling and on inhibition of nuclear translocation of β-catenin, while combined inhibition of TLR4 and GSK-3β restored β-catenin activity and osteogenic differentiation to near-control levels (Figure 6C). Because endotoxin contamination could confound TLR4-associated responses, the heme preparation was additionally assessed using a chromogenic LAL assay. The heme-only response was below the lowest endotoxin standard tested. These findings suggest that TLR4 contributes to the anti-osteogenic effects of heme and support the concept that innate immune signaling directly intersects with molecular pathways governing osteoblast differentiation [48].

Importantly, TLR4 signaling in bone appears highly context-dependent [30,48,49]. Whereas physiological TLR4 signaling may contribute to skeletal adaptation and remodeling, persistent activation by endogenous danger signals is generally associated with impaired osteogenesis and pathological bone remodeling [49]. Our findings indicate that extracellular heme belongs to this latter category and identify a specific osteoimmunological mechanism through which tissue injury and hemorrhage may negatively influence bone formation [7,8,29,49].

The in vivo findings strengthen the physiological relevance of the proposed mechanism [50]. Maternal heme exposure reduced alkaline phosphatase activity in multiple craniofacial ossification centers during active skeletal development, indicating that the anti-osteogenic effects of extracellular heme are not restricted to isolated cell culture systems. Although the developmental model does not directly address postnatal bone regeneration, the concordance between the in vitro and in vivo findings supports the conclusion that extracellular heme can negatively regulate osteogenesis in a complex biological environment [10]. Interestingly, pharmacological inhibition of Nrf2 further aggravated the suppressive effects of heme on osteogenic gene expression, indicating that endogenous cytoprotective pathways partially counteract heme-induced osteogenic dysfunction (Figure S2). Nrf2 is a master regulator of antioxidant defense mechanisms and an important component of cellular adaptation to heme stress through induction of genes such as HO-1 [51,52]. Although the present study did not directly assess activation of the Nrf2-HO-1 axis [53,54], our findings suggest that endogenous Nrf2 signaling may function as a compensatory response limiting the detrimental effects of extracellular heme on osteoblast differentiation (Figure 6D). Future studies should determine whether activation of Nrf2 or enhancement of physiological heme-scavenging systems such as hemopexin can preserve osteogenesis under heme-rich conditions [12,13,53,54,55,56].

The exposure levels used in the present study should primarily be interpreted within a mechanistic proof-of-concept framework, although the in vitro concentration is within a pathophysiologically plausible range. Circulating plasma heme concentrations of approximately 0–20 μM at steady state and 20–40 μM during vaso-occlusive episodes have been reported in patients with sickle cell disease, while a recent study directly measuring unbound cell-free heme reported mean concentrations of approximately 10–23 μM across different SCD subgroups [57,58]. Notably, in an experimental canine model of subarachnoid hematoma, total hemin concentrations reached 390 ± 247 μM in clotted material 7 days after blood deposition, whereas freshly clotted blood contained no detectable hemin [24]. In vitro, 10 μM heme was selected based on dose–response assessment of ALP activity together with cell viability and was therefore used for the subsequent mechanistic experiments. Nevertheless, circulating heme is distributed among protein-bound, membrane-associated, and unbound pools; therefore, the biological availability of circulating heme cannot be assumed to be directly equivalent to that of heme added to culture medium. For the in vivo experiments, the maternal heme doses of 30 and 75 μmol/kg body weight were adopted from our previously published murine pregnancy model [10]. Thus, the experimental exposures used here should not be interpreted as direct quantitative surrogates for heme levels in a specific human disease.

The translational implications of these findings may be considerable [11,59,60]. Our data identify extracellular heme as a potential regulator of skeletal regeneration and suggest that the balance between heme generation and heme detoxification may influence osteogenic outcomes following injury. These observations raise the possibility that therapeutic strategies aimed at limiting extracellular heme signaling or enhancing endogenous heme-scavenging pathways could help preserve bone formation in inflammatory and hemorrhagic conditions [15,55]. However, these concepts require validation in clinically relevant models of fracture repair and skeletal regeneration.

In summary, our findings support a model in which heme suppresses osteogenesis through TLR4-associated signaling, reduced AKT activity, increased GSK-3β activation, impaired β-catenin signaling, and subsequent inhibition of osteogenic differentiation. At the same time, endogenous Nrf2-dependent responses may partially counteract these detrimental effects (Figure 6).

Several limitations should be considered. The mechanistic studies were performed primarily in MC3T3-E1 cells and require validation in primary murine and human osteoprogenitor populations. In addition, the involvement of TLR4 and GSK-3β was established predominantly using pharmacological approaches, and complementary genetic studies will be required to confirm causality. Direct assessment of bone volume, architecture, and fracture repair was beyond the scope of the present study and should be addressed in future investigations. The contribution of iron-mediated stress was not separately assessed and should be examined using heme-free soluble iron controls. Finally, bioavailable extracellular heme was not directly characterized under our culture conditions. We cannot formally exclude contributions of serum-dependent heme binding and redistribution under culture conditions. Future studies using hemopexin-mediated scavenging will be required to determine the contribution of bioavailable extracellular heme.

## 4. Materials and Methods

### 4.1. Reagents

Hemin (ferriprotoporphyrin IX chloride; Frontier Specialty Chemicals, Logan, UT, USA; catalog no. H651-9) was freshly prepared and dissolved in NaOH as previously described [59]. Because alkaline dissolution results in ferric heme species that may include hematin-like forms, the term “heme” is used throughout our manuscript as a general descriptor of extracellular ferric heme preparations [12]. Briefly, hemin was dissolved in 0.1 M NaOH containing Tris base, diluted in Milli-Q water, adjusted to pH 7.8–8.2 with HCl, and filter-sterilized (0.22 µm). Heme solutions were protected from light and used immediately after preparation. Endotoxin contamination in a 20 μM heme was assessed using the ToxinSensor™ Chromogenic LAL Endotoxin Assay Kit (Cat. No. L00350; GenScript, Piscataway, NJ, USA). Endotoxin standards ranging from 0.010 to 0.100 EU/mL were used for calibration, and a 0.05 EU/mL endotoxin was used as an endotoxin-positive control. The selective Toll-like receptor 4 (TLR4) inhibitor TAK-242 [61] (Resatorvid; MedChemExpress, Monmouth Junction, NJ, USA; Cat. No. HY-11109; purity ≥ 99%) and the Nrf2 inhibitor ML385 (MedChemExpress, Monmouth Junction, NJ, USA; Cat. No. HY-100523; purity ≥ 99%) were dissolved in dimethyl sulfoxide (DMSO), filter-sterilized, aliquoted, and stored at −80 °C. Lithium chloride (LiCl; Sigma-Aldrich, St. Louis, MO, USA) was used as a pharmacological inhibitor of GSK-3β. The final DMSO concentration in cell cultures did not exceed 0.01% (*v*/*v*). Vehicle controls were included; as no differences were observed between untreated and vehicle-treated groups, untreated controls were used as the reference for statistical analyses.

### 4.2. Cell Culture and Osteogenic Differentiation

The mouse pre-osteoblastic cell line MC3T3-E1 (ATCC, Manassas, VA, USA) was cultured in α-minimal essential medium (α-MEM; Gibco, Life Technologies Corporation, Grand Island, NY, USA) supplemented with 10% fetal bovine serum (FBS; Gibco, Life Technologies Limited, Paisley, UK), 100 U/mL penicillin, and 100 μg/mL streptomycin (Gibco, Life Technologies Corporation, Grand Island, NY, USA). Cells were maintained at 37 °C in a humidified atmosphere with 5% CO_2_. For osteogenic differentiation, confluent cultures were switched to osteogenic medium consisting of α-MEM supplemented with 10 mM β-glycerophosphate, 10 nM dexamethasone, and 50 μg/mL ascorbic acid (vitamin C). Cells were maintained under osteogenic conditions for up to 21 days and treated with heme (10 μM) and/or LiCl (1 mM), with TAK-242 (1 μM) or ML385 (10 μM) administered 2 h before heme exposure, as indicated. Unless stated otherwise, experiments were performed using three independent biological replicates.

### 4.3. Alkaline Phosphatase (ALP)-Activity

Osteogenic differentiation was assessed by measuring ALP activity at day 3. MC3T3-E1 cells were seeded at 5 × 10^3^ cells/well in 48-well plates (500 µL/well; *n* = 4 per condition) and cultured in osteogenic medium with heme (10 μM) and/or LiCl (1 mM), with TAK-242 (1 μM) administered 2 h before heme exposure, as indicated, for 3 days. Cells were washed with phosphate-buffered saline (PBS), stored at −20 °C and lysed by 2 freeze–thaw cycles in 1 mM Tris buffer. ALP activity was measured using a colorimetric assay based on p-nitrophenyl phosphate hydrolysis. Briefly, 80 µL of each lysate or standard (0–25 nM serial dilutions of 4-dinitrophenol) was mixed with 20 µL assay buffer (0.5 M 2-amino-2-methyl-1-propanol) and 100 µL substrate solution (p-nitrophenyl phosphate) in a 96-well plate. The reaction mixture was incubated at 37 °C for 60 min and terminated by adding 20 µL of 0.3 M NaOH. Absorbance was measured at 405 nm using a Synergy HTX microplate Reader (BioTek, Winooski, VT, USA). DNA content was quantified using the QuantifluorTM dsDNA kit (Promega, Madison, WI, USA) according to the manufacturer’s instructions. ALP activity was expressed as nmol p-nitrophenyl phosphate/h/ng DNA.

### 4.4. Cell Viability

Cell viability was assessed using the Cell Counting Kit-8 (CCK-8; Sigma, St. Louis, MO, USA). Cells were seeded at 1 × 10^4^ cells/well in 96-well plates (*n* = 5 per condition) and treated with heme (10 μM) for 12, 24, 48, or 72 h. At each time point, cells were incubated with CCK-8 reagent (1:10 dilution in osteogenic medium; CCK-8; Sigma, St. Louis, MO, USA) for 2 h at 37 °C. Absorbance was measured at 450 nm using a Synergy HTX microplate Reader (BioTek, Winooski, VT, USA). Cell viability at each time point was expressed as fold change relative to the corresponding untreated control.

### 4.5. RNA Isolation and Quantitative RT-PCR

MC3T3-E1 cells (2 × 10^4^ cells/well, 12-well plates) were cultured under osteogenic conditions with heme (10 μM) and/or LiCl (1 mM), with TAK-242 (1 μM) administered 2 h before heme exposure, as indicated. Axin2, Lef1, Ccnd1, and c-Myc were assessed at 24 h; Runx2 and Sp7 at day 3; Alpl and Col1a1 at day 7; and Bsp and Bglap at day 10. ML385 (10 μM) was administered 2 h before heme exposure, with Runx2 assessed at day 3. Total RNA was isolated using the RNeasy Mini Kit (Qiagen, Hilden, Germany) according to the manufacturer’s instructions, and 1 µg RNA was reverse transcribed using the iScriptTM Reverse Transcription Supermix system (Bio-Rad, Hercules, CA, USA). Quantitative real-time PCR (RT-qPCR) was performed using SYBR Green Supermix (Bio-Rad) on a CX96 Real-Time PCR Detection System (Bio-Rad). Cycling conditions consisted of an initial denaturation at 95 °C for 3 min, followed by 39 cycles of 95 °C for 15 s and 60 °C for 30 s. Relative gene expression levels were normalized using the geometric mean of the reference genes Gapdh, Actb (β-actin), and 18S rRNA and calculated using the 2^−ΔΔCt^ method as described previously [62,63]. Primer sequences are listed in Table 1. 

### 4.6. Western Blotting

MC3T3-E1 cells were seeded at 1 × 10^5^ cells/well in 6-well plates and treated with heme (10 μM) and/or LiCl (1 mM), with TAK-242 (1 μM) administered 2 h before heme exposure, as indicated, for 24 h before protein extraction. Total cellular protein was extracted using radioimmunoprecipitation assay (RIPA) lysis buffer supplemented with protease and phosphatase inhibitors. Equal amounts of protein were separated by 12% SDS-PAGE and transferred onto 0.45 µm nitrocellulose membranes.

Membranes were blocked in 5% skim milk (ELK, Campina, Amersfoort, The Netherlands) in Tris-buffered saline containing 0.1% Tween-20 (TBST) and incubated overnight at 4 °C with primary antibodies against AKT (1:2000; Cell Signaling Technology, Danvers, MA, USA), phosphorylated AKT (Ser473; 1:2000; Cell Signaling Technology, Danvers, MA, USA), β-catenin (1:5000; Abcam, Cambridge, UK), active β-catenin (non-phospho; 1:2000; Cell Signaling Technology, Danvers, MA, USA), GSK-3β (1:2000; Proteintech, Rosemont, IL, USA), phospho-GSK-3β (Ser9; 1:5000; Proteintech, Rosemont, IL, USA), GAPDH (1:50,000; Proteintech, Rosemont, IL, USA) and β-actin (1:1000; Cell Signaling Technology, Danvers, MA, USA).

After incubation with horseradish peroxidase-conjugated secondary antibodies (1:5000; anti-mouse or anti-rabbit IgG) for 2 h at room temperature, and washing five times with TBST, bands were visualized using enhanced chemiluminescence (ECL) (GE Healthcare, UK; Cat. No. 16961643). Densitometric analysis was performed using ImageJ/Fiji software (version 2.16.0/1.54p).

### 4.7. Immunofluorescence and Quantification of β-Catenin Nuclear Localization

MC3T3-E1 cells (1 × 10^4^ cells/well) were cultured on chamber slides (Millicell EZ, Merck Millipore, Billerica, MA, USA) and treated with heme (10 μM), LiCl (1 mM), and/or TAK-242 (1 μM), as indicated, for 6 h; TAK-242 was administered 2 h before heme exposure. Cells were then fixed with 4% paraformaldehyde, permeabilized (0.5% Triton X-100), and blocked (100 mM glycine in blocking buffer including PBS containing 2% (*w*/*v)* BSA, 2% (*v*/*v*) NDS, 0.1% (*v*/*v*) Triton X-100, and 0.05% (*v/v*) Tween-20). Cells were incubated with anti-β-catenin antibody (1:1000; Abcam, Cambridge, UK) overnight at 4 °C, followed by incubation with Alexa Fluor 488-conjugated secondary antibody (1:200) for 1 h at room temperature in the dark. Nuclei were counterstained with DAPI (0.4 µg/mL). Fluorescence images were acquired using identical exposure settings for all experimental conditions. β-catenin nuclear localization was quantified as the nuclear-to-cytoplasmic fluorescence intensity ratio using ImageJ/Fiji software (version 2.16.0/1.54p). Images were taken with a fluorescence microscope (ZEISS Axio Imager.Z2 microscope, Carl Zeiss Microscopy GmbH, Jena, Germany; ZEN blue, version 3.3.89.0000) at 20× magnification and were converted to 8-bit grayscale. The same settings of brightness, light exposure and saturation were used for all images. Regions of interest (ROIs) were manually selected for the nucleus and the whole cell, with nuclear regions identified by DAPI staining and whole-cell boundaries determined by cell morphology. Fluorescence intensity was measured for each ROI. Cytoplasmic fluorescence intensity was calculated as total cellular intensity minus nuclear intensity, and the nuclear-to-cytoplasmic ratio was determined accordingly.

### 4.8. Calcium Quantification and Mineralization 

#### 4.8.1. Calcium Deposition

MC3T3-E1 cells were seeded at 5 × 10^3^ cells/well in 48-well plates (500 µL/well; *n* = 4 per condition). Calcium deposition was quantified after 14 days of osteogenic differentiation with heme (10 μM) and the indicated pharmacological treatments. Mineralized matrix was extracted with 0.5 M acetic acid, followed by colorimetric measurement using a working solution consisting of 5% 14.8 M ethanolamine (Merck)/boric acid buffer (BOOM; pH 11), 5% ortho-cresolphthalein complexone (Merck), and 2% hydroxyquinoline (Sigma). Absorbance was measured at 570 nm using a Synergy HTX microplate Reader (BioTek, Winooski, VT, USA).

#### 4.8.2. Alizarin Red S Staining

To assess extracellular matrix mineralization, cells (2 × 10^4^ cells/well, 12-well plates) were cultured under osteogenic conditions with heme (10 μM) and the indicated pharmacological treatments for 21 days, fixed in 10% formalin, and stained with 40 mM Alizarin Red S solution (Sigma-Aldrich, St. Louis, MO, USA; pH 4.1–4.2; 1 mL per well) for 20 min at RT. Images were acquired using a bright-field microscopy.

### 4.9. In Vivo Analysis of Craniofacial Development

All animal procedures were approved by the Board for Animal Experiments of the Radboud University Nijmegen (Ethical permission # RU-DEC 2012-166) and conducted in accordance with institutional and national guidelines. The plugged animals were transported to our animal facility and housed under specific pathogen-free housing conditions with a 12 h light/dark cycle and ad libitum access to water and powdered rodent chow (Sniff, Soest, The Netherlands) as described in detail earlier [10]. The animals could acclimatize for at least 1 week before the start of the experiment. All pregnant mice were randomly assigned to control or experimental groups by drawing lots. Pregnant CD1 mice (Envigo, Venray, The Netherlands) received intraperitoneal injections of heme (0, 30, or 75 µmol/kg) as described previously in detail [10]. Fetuses were harvested at E16. Coronal sections of fetal heads from these fetuses [10] were stained with hematoxylin and eosin (H&E) for morphological assessment of palatal development and classified according to established developmental stages [64]. Osteogenic activity was evaluated by ALP staining as previously described [65]. A minimum of three embryos per experimental group and multiple sections per embryo were analyzed. The developmental study was intended as proof-of-concept and future studies with larger cohorts will be required to determine effects on craniofacial bone volume and architecture.

### 4.10. Quantification of ALP Activity In Vivo

ALP activity in coronal sections was visualized using NBT/BCIP staining. All sections were processed together in the same staining container under identical conditions. All images were acquired using identical microscope settings and analyzed using identical threshold parameters. The workflow is shown in Figure 5B. Quantitative analysis was performed using ImageJ/Fiji software (version 2.16.0/1.54p). Regions of interest (ROIs) corresponding to the frontal, nasal, palatal and mandibular regions were manually outlined according to anatomical boundaries. For each ROI, a uniform color threshold was applied to identify the blue-purple NBT/BCIP-positive staining. The ALP-positive area fraction, defined as the ratio of ALP-positive area to the total ROI area, was automatically calculated using ImageJ/Fiji (version 2.16.0/1.54p) and used as the quantitative indicator of ALP activity. One representative coronal section was analyzed per mouse, and each mouse was considered one independent biological replicate (*n* = 3 mice per group).

### 4.11. Statistical Analysis

Data are presented as mean ± standard deviation (SD). Statistical analyses were performed using GraphPad Prism 9.5.0 (GraphPad Software, San Diego, CA, USA). Data distribution was assessed using the Shapiro–Wilk test, and homogeneity of variance was evaluated using the Brown–Forsythe test. Comparisons between two groups were performed using an unpaired two-tailed Student’s *t*-test. Comparisons among three or more groups were analyzed using one-way analysis of variance (ANOVA) followed by Tukey’s multiple comparisons test. Cell viability data from CCK-8 assays were analyzed separately at each time point using multiple unpaired two-tailed *t*-tests with Holm–Šídák correction for multiple comparisons. Sample sizes were based on previous studies employing similar osteogenic endpoints. A *p* value below 0.05 (*p* < 0.05) was considered statistically significant.

## 5. Conclusions

In conclusion, our findings identify extracellular heme as an osteogenesis suppressor and establish a mechanistic link between heme-associated danger signaling and impaired bone formation. Extracellular heme inhibits osteoblast differentiation through TLR4-dependent suppression of the AKT/GSK-3β/β-catenin pathway, resulting in reduced canonical Wnt activity and impaired matrix mineralization. These findings expand current concepts in osteoimmunology [7,8] by identifying a heme-derived DAMP as a regulator of osteogenic cell fate and suggest that modulation of heme signaling may represent a potential strategy to improve bone regeneration in inflammatory, hemorrhagic and other heme-rich pathological environments.

## Figures and Tables

**Table 1:** Primer sequences used for RT-qPCR analysis of mouse genes.

Gene	Forward Primer (5′-3′)	Reverse Primer (5′-3′)
*Runx2*	CGGACGAGGCAAGAGTTTCA	GGATGAGGAATGCGCCCTAA
*Sp7*	CCTACCCAGGAAGAAGCTCAC	TGGTTTTGGGGGCTGAAAGG
*Alpl*	CCAGCAAGAAGAAGCCTTTG	AACCCAGACACAAGCATTCC
*Col1a1*	AAGGTGCCAATGGTGCTC	ACCAGTGTCTCCTTTGTTGC
*Bsp*	GTTTGAAGTCTCCTCTTCCTCC	TTTATCCTCCTCTGAAACGGT
*Bglap*	CCGGGAGCAGTGTGAGCTTA	CCATACTGGTCTGATAGCTC
*Axin2*	GGTTCCGGCTATGTCTTTGC	CAGTGCGTCGCTGGATAACTC
*c-Myc*	GGTTTGCCTCTTCTCCACAG	TCCTGTACCTCGTCCGATTC
*Lef1*	TATGAACAGCGACCCGTACA	TCGTCGCTGTAGGTGATGAG
*Ccnd1*	TTTCTTTCCAGAGTCATCAAGTGT	TGACTCCAGAAGGGCTTCAA
*Gapdh*	GGCAAATTCAACGGCACAGT	CCTCACCCCATTTGATGTTAGTG
*Actb*	CGGTTCCGATGCCCTGAGGCTCTT	CGTCACACTTCATGATGGAATTGA
*18S rRNA*	GTAACCCGTTGAACCCCATT	CCATCCAATCGGTAGTAGCG

## Data Availability

The original contributions presented in this study are included in the article/Supplementary Material. Further inquiries can be directed to the corresponding authors.

## Supplementary Materials

The following supporting information can be downloaded at: https://www.mdpi.com/article/10.3390/ijms27209305/s1.

## Abbreviations

The following abbreviations are used in this manuscript:

TLR4	Toll-like receptor 4
GSK-3β	glycogen synthase kinase-3β
Hpx	hemopexin
Nrf2	nuclear factor erythroid 2-related factor 2
HO-1	heme oxygenase-1
