# Supplementary material for: Heme-Mediated Danger Signaling Suppresses Osteogenesis Through TLR4-Dependent Inhibition of β-Catenin Signaling
Source: Int J Mol Sci. 2026 Oct 20;27(20):9305. doi: 10.3390/ijms27209305 (PMC13705325; doi:10.3390/ijms27209305)
Supplement: Supplementary file 1 [file ijms-27-09305-s001.zip › ijms-4592390-supplementary.pdf]

International Journal of
Molecular Sciences

MDPI

Supplementary Materials:

The following supporting information can be downloaded at: https://www.mdpi.com/article/doi/s1, Figure S1: Limulus amebocyte lysate (LAL) analysis showed that heme (20 μM) had an absorbance (A545) of 0.0503 ± 0.0005, which was below the response of the lowest endotoxin standard tested (0.010 EU/mL; A545 = 0.0600) (Supplementary Figure S1).

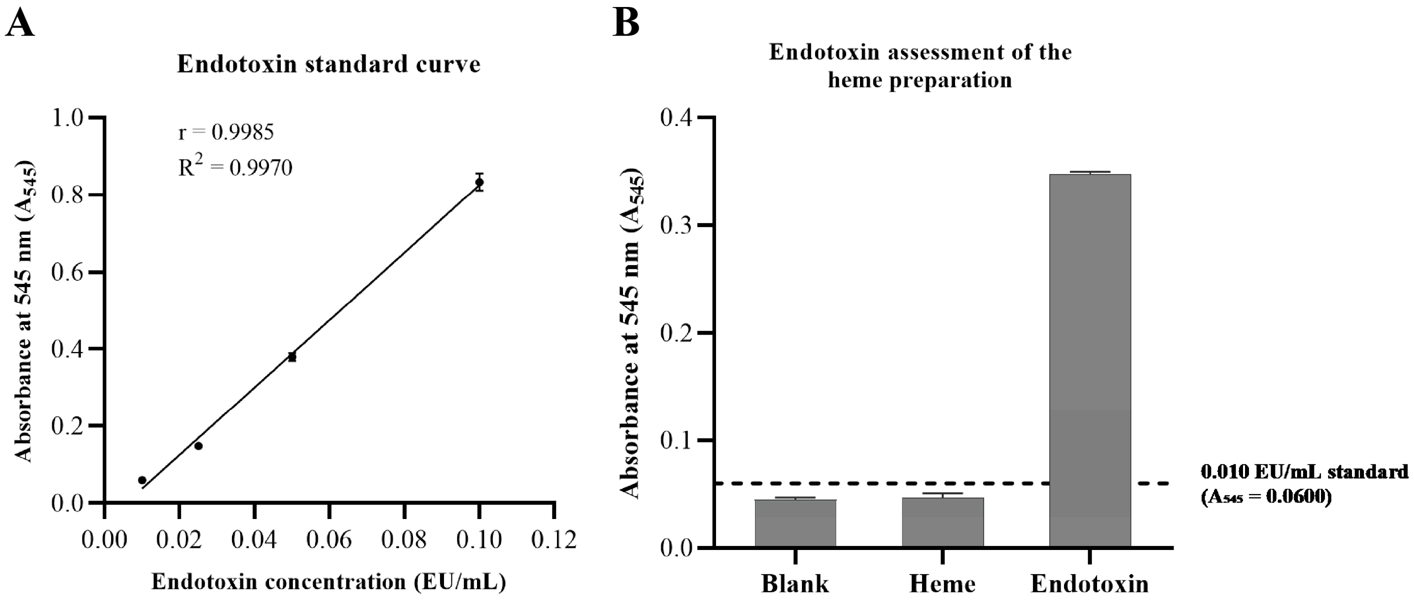

Supplementary Figure S1. Endotoxin assessment of the heme preparation. (A) Endotoxin standard curve over 0.010–0.100 EU/mL (r = 0.9985, R^2 = 0.9971). Standards at 0.010, 0.025, 0.050 and 0.100 EU/mL were measured in four technical replicates. (B) Chromogenic LAL assay using blank (LAL Reagent Water), heme (20 μM), and endotoxin (0.05 EU/mL; positive control). Bars show mean ± SD of four technical replicate measurements. The dashed line indicates the 0.010 EU/mL standard (A_{545} = 0.0600).

The following supporting information can be downloaded at: https://www.mdpi.com/article/doi/s2, Figure S2: Nrf2 signaling partially protects against heme-induced osteogenic suppression. To explore whether endogenous cytoprotective signaling modulates the osteogenic response to heme, cells were treated with the Nrf2 inhibitor ML385. Pharmacological inhibition of Nrf2 further decreased expression of the osteogenic transcription factor Runx2 in heme-treated cultures (Supplementary Figure S2), indicating that Nrf2 inhibition aggravated the response and suggesting a potential protective role for endogenous Nrf2 signaling. These findings suggest that extracellular heme simultaneously activates detrimental signaling pathways, including TLR4, while eliciting adaptive Nrf2-dependent responses that limit osteogenic dysfunction.

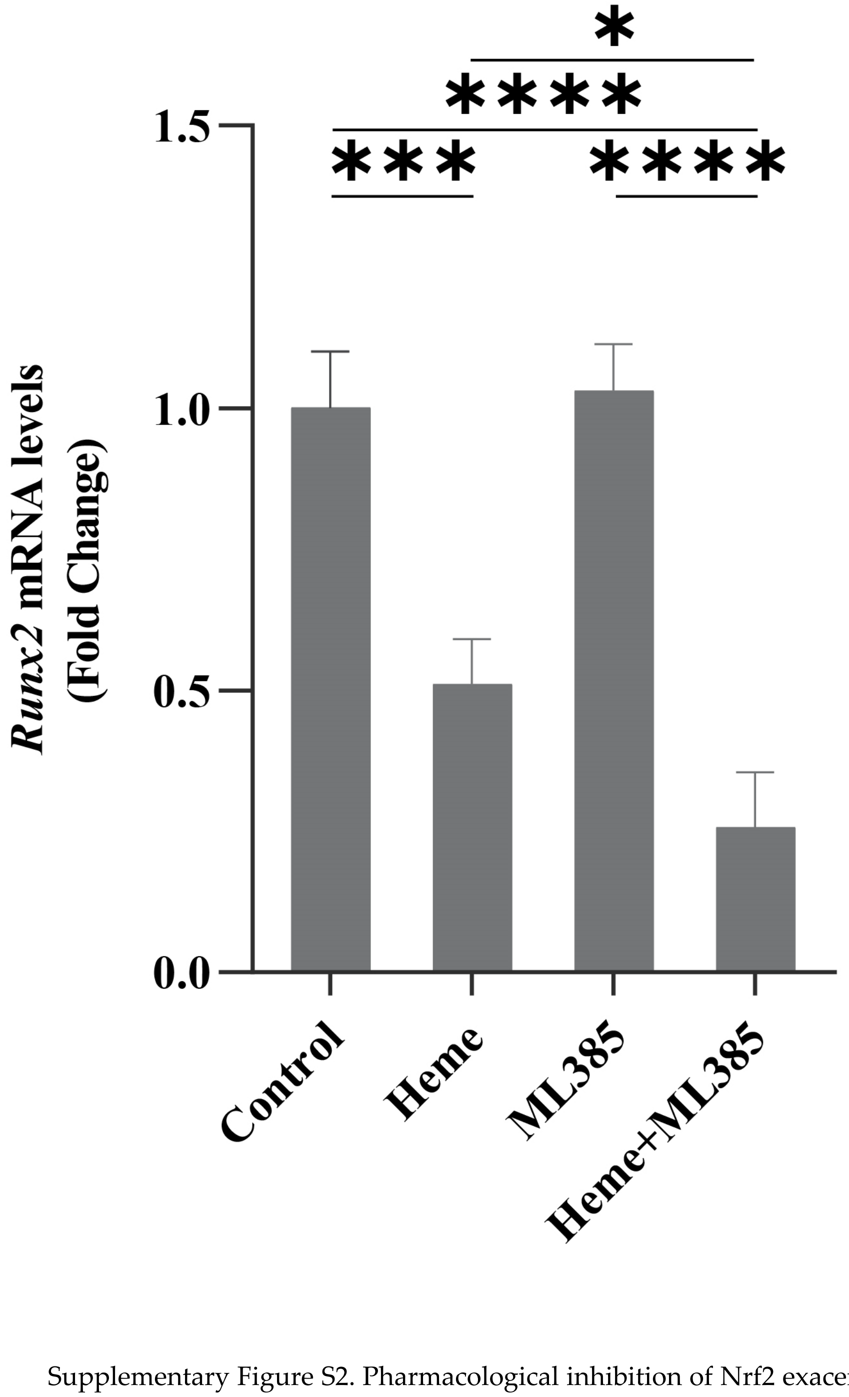

Supplementary Figure S2. Pharmacological inhibition of Nrf2 exacerbates heme-induced suppression of osteogenic gene expression. MC3T3-E1 cells were pretreated with the Nrf2 inhibitor ML385 (10 μM) for 2 h before exposure to heme (10 μM). Runx2 mRNA expres-

sion was measured by RT-qPCR after 3 days of osteogenic differentiation. Data are presented as mean ± SD from three independent experiments. * $p < 0.05$, *** $p < 0.001$, **** $p < 0.0001$.
